# Selective caries removal and management of exposed pulp in fully developed and immature teeth with reversible pulpitis: a questionnaire-based study in Greece

**DOI:** 10.1007/s40368-025-01024-7

**Published:** 2025-05-27

**Authors:** E. Babasidou, G. Papaemmanouil, A. Pantelidou, A. Fardi, K. Kodonas, C. Gogos

**Affiliations:** 1 Department of Endodontology, School of Dentistry, Aristotle University of Thessaloniki, Thessaloniki, Greece; 2 Department of Dentoalveolar Surgery, Surgical Implantology and Radiology, School of Dentistry, Aristotle University of Thessaloniki, Thessaloniki, Greece

**Keywords:** Deep caries lesions, Greek dentists, Reversible pulpitis, Root canal treatment, Vital pulp treatment

## Abstract

**Purpose:**

Guidelines recommend implementing selective or total caries removal for managing deep carious lesions without discriminating between fully developed and immature teeth. This questionnaire-based study aimed to explore the perspectives of Greek dentists regarding the management of deep caries and exposed pulp in immature and fully developed teeth with reversible pulpitis.

**Methods:**

The questionnaire presented two cases: one of a fully developed permanent tooth and one of an immature permanent tooth, both with deep caries and clinical signs of reversible pulpitis. Photographs, radiographs, and clinical symptoms were provided to assess dentists’ treatment strategy preferences. Data analysis was conducted using SPSS 28, Chi-square, Fisher’s exact tests and logistic regression analysis with significance set at *p* < 0.05.

**Results:**

A total of 453 dentists responded. More than half of the respondents preferred total caries removal for mature teeth. MTA and other bioceramics emerged as the favored materials for indirect and direct pulp capping. In the case of the immature tooth, 44% of the respondents shifted from the total to selective caries removal treatment option. However, there was a discernible shift towards more aggressive vital pulp treatment options, like pulpotomy (26%).

**Conclusion:**

Treatment preferences are influenced by the tooth developmental status, vary significantly and there is no clear preference for a more conservative approach to preserving as much healthy pulp tissue as possible.

## Introduction

Deep caries lesions (DCLs) are defined as those reaching radiographically into the pulpal third of the dentin without pulp exposure (Innes et al. 2016). Clinical management of DCLs varies greatly among dentists in terms of diagnostic criteria, the approach to carious tissue removal (selective or total caries removal), and methods for caries detection. The diagnosis of dental pulp and periapical tissue pathology is based on a variety of clinical symptoms including the tooth’s reaction to temperature changes, the spontaneity of the pain, any sleep disturbances it may cause, and clinical signs revealed during examination like tooth reaction to thermal and electric pulp testing. According to the terminology of the American Association of Endodontists (AAE) reversible pulpitis is a clinical diagnosis based on subjective and objective findings indicating that inflammation could resolve, and the pulp could return to normal (Glickman 2009). The diagnosis of reversible pulpitis is primarily determined by the intensity and duration of pain triggered by thermal stimuli. In such cases, applying a cold stimulus results in a brief intense pain response that does not persist longer than in a control tooth. On the contrary, spontaneous, radiating, lingering pain is indicative of irreversible pulpitis. It is important to note that these diagnostic techniques are subjective and relative, offering only general clinical guidance (ESE 2019, AAE 2021).

Fusayama et al. classified two areas within a carious lesion: an outer caries-infected layer and an inner caries-affected layer (Fusayama et al. 1966). Deep caries can be removed through various methods according to the International Caries Detection and Assessment System (Gugnani et al. 2011). These methods include total or non-selective caries removal, and selective caries removal which can be performed in one or two steps (stepwise technique). Selective removal involves removing carious tissue to soft or firm dentin on the pulpal aspect of the cavity. Carious tissue is removed from the peripheral walls to expose hard dentin providing the necessary sound dentin borders for an adequate seal with a well-adapted restoration that is ideally bonded to sound tooth tissue at the cavity margins. Total caries removal involves completely removing soft and firm carious dentin from the periphery and central aspects of the cavity until reaching hard dentin (ESE 2019). Completely excavating deep caries down to hard dentin has a significant risk of pulp exposure (Ricketts et al. 2013).

It has been widely documented that there is no consistency in the dental community regarding the most suitable treatment for DCLs management (ESE 2019, AAE 2021). According to the position statement of the European Society of Endodontology (ESE) (ESE 2019) and International Caries Consensus Collaboration (ICCC) (Schwendicke et al. 2016), selective caries removal may be considered a viable option for managing DCLs in teeth presenting signs and symptoms of reversible inflammation. This non-invasive approach has been supported by randomized clinical trials showing that in cases of selective caries removal, pulp exposure was avoided and teeth presented higher success rates (Bjørndal et al. 2017; Maltz et al. 2018). On the contrary, the AAE supports total removal of caries even in cases where pulp exposure is likely to occur, to eliminate infected dentin and directly evaluate pulp tissue by visualization (AAE 2021). Obviously, the ESE and AAE recommendations appear to be contradictory. Moreover, both treatment strategies do not appear to distinguish between cases of fully developed or immature teeth. This lack of distinction may be attributed to the limited evidence in literature for immature teeth. It has been recently evidenced that VPT modalities present similar success rates in immature teeth while less-invasive procedures seem to be advantageous for mature teeth (Tong et al. 2022; Barros et al. 2020). However, less-invasive treatment options do not seem to have been implemented into clinical practice (Schwendicke and Gostemeyer 2016). Although essential preventive measures for caries disease, such as the implementation of public or individual preventive programmes have been widely utilized, a well-defined evidence-based, and aetiology-related dental disease management strategy has yet to be implemented (Kühnisch et al. 2016).

Due to the lack of clear guidelines, it is important to investigate the preferred treatment modalities within the dental community. To the best of the authors’ knowledge, no studies have been published in Greece that provide current information on dentists’ management of DCLs in fully developed or immature teeth with reversible pulpitis. The aim of this study is to investigate the perspectives of Greek dentists regarding the management of deep caries and exposed pulp in immature and fully developed teeth with reversible pulpitis.

## Methods

This descriptive, cross-sectional study employed an online questionnaire to investigate Greek dentists’ attitudes towards adopting conservative methods for caries removal and pulp capping procedures in immature and fully developed teeth with reversible pulpitis. The guidelines from the ESE (ESE 2019), ICCC (Schwendicke et al. 2016) and AAE (AAE 2021) international organizations were taken into consideration for the design of the questionnaire. STROBE checklist for cross-sectional studies was also used to standardize the reporting quality of this study (Elm et al. 2007). The Research Ethics Committee of the Dental School granted ethical approval for this survey (No:219). The questionnaire was distributed through multiple channels to maximize reach and response rates across Greece, ensuring a wide geographical representation. It was administered using EUSurvey Forms (https://ec.europa.eu/eusurvey) to ensure the anonymity of the respondents and the protection of their personal data and shared via email and professional dental networks. Additionally, the survey link was disseminated through social media, including dental forums, LinkedIn and Facebook groups dedicated to Greek dental professionals on a nationwide scale. Furthermore, the questionnaire was promoted during online dental conferences and webinars, encouraging participation from attendees. Due to the exclusive use of digital distribution methods, the exact number of dentists approached could not be accurately measured. However, when considering the total number of members in the dental community who were approached by online networks, as long as the participants of the webinars and online conferences where the link was distributed, a minimum number of 3000 dentists were expected to have been finally reached.

Written informed consent to participate was not directly obtained but inferred by the completion of the questionnaire in the survey. The survey was conducted in two stages: the first stage involved formulating, designing, and validating the content. Ten experienced dentists initially completed the questionnaire for content validation. Five investigators then modified them accordingly and finalized them to ensure that the acquired data accurately represented clinical practice. The second stage included distributing the validated questionnaire to dentists from January 2023 to May 2023.

**Fig. 1:**
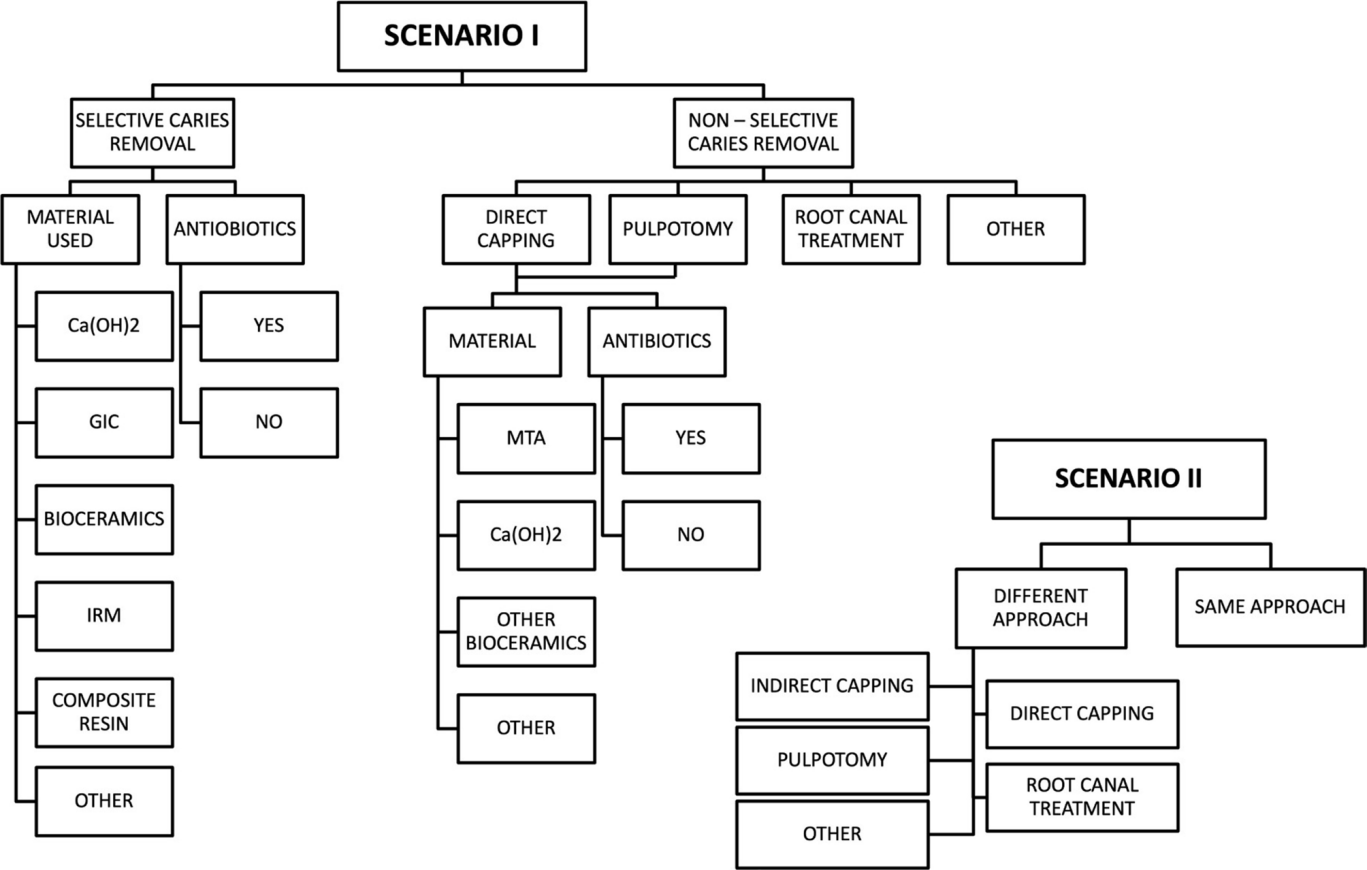
A decision flow chart that visualizes the potential treatment options of the questionnaire. The sequence of steps and flow direction represent the selection of treatment modality, capping materials or the decision to use antibiotics by the respondents

The web-based questionnaire consisted of two parts. The first part of the survey inquired about respondents’ profiles including demographics, years of clinical experience, working environment, educational status, and methods of continuous education. The second part presented two different clinical scenarios involving vital permanent mature and immature teeth with deep carious lesions. High-resolution photographs taken by the operating microscope, radiographs, and a description of symptoms that led to the diagnosis of reversible pulpitis were provided. The questionnaire asked dentists about their preferences for treatment strategies, procedural choices, materials used and antibiotic prescription (shown in Fig. 1). It contained a combination of open-ended questions to encourage manual input and multiple-choice questions.

### Clinical cases

**Fig. 2:**
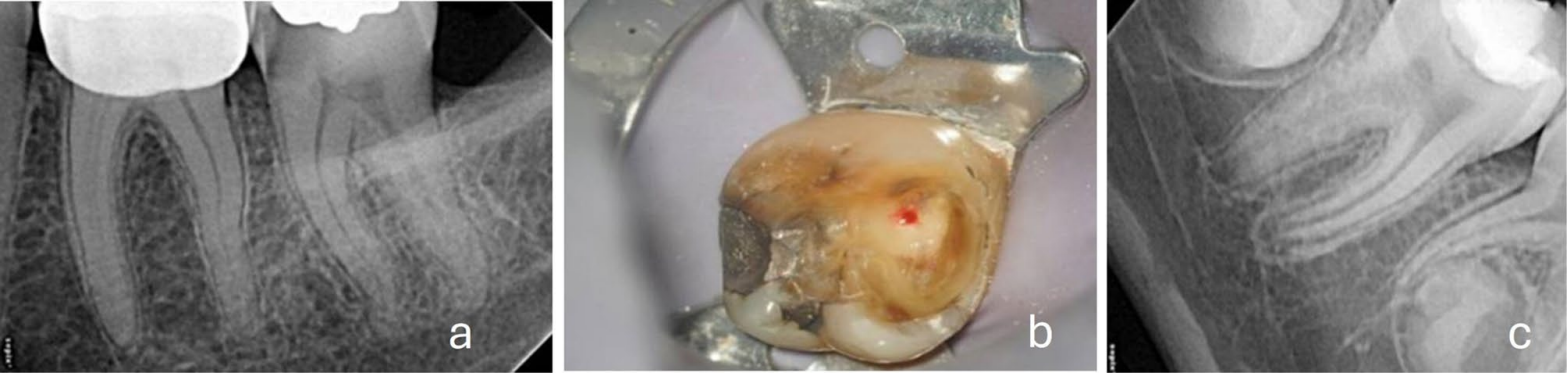
**a** Scenario I: radiograph of tooth #37 showing a deep caries lesion, fully formed apices and normal periapical tissues. **b** Scenario I. Operative microscope photograph of pulp exposure following complete excavation of carious dentine of tooth #37 presenting signs of reversible pulpitis. **c** Scenario II: radiograph of tooth #46 showing a deep caries lesion, immature apices and normal periapical tissues

The first scenario included a 22-year-old female patient with an asymptomatic deep caries lesion on tooth #37. The sensibility test revealed an increased response to cold stimulus, which subsided at the same time compared to a control tooth. Percussion and palpation tests were negative. Radiographic examination showed a lesion reaching the inner quarter of dentin with no signs of periapical radiolucency (shown in Fig. 2a). Participants were informed that during caries removal, the carious dentin can be completely removed from the axial walls with the potential of pulp exposure. The study recorded the preferred materials for indirect pulp capping for those preferring selective caries removal. The questionnaire did not discriminate options between one- or two-step (stepwise) caries removal method to clearly emphasize on the main dilemma which is the selection between total or selective caries removal. For those selecting total caries removal a scenario involving the management of a carious pulp exposure was presented and their treatment preferences were recorded; direct pulp capping, pulpotomy or root canal treatment (shown in Fig. 2b). During the first two vital pulp treatment options, the dentists were inquired about the preferred materials.

The second scenario included an 8-year-old male patient presenting an asymptomatic deep caries lesion on tooth #46 (shown in Fig. 2c). Sensibility test revealed an increased response to cold stimulus, but the response subsided at the same time compared to a control tooth. Percussion and palpation were negative. The periapical tissues appeared normal. Similar to scenario I, during caries removal, the carious dentin could be completely removed from the axial walls. However, the complete removal of dentin on the pulpal aspect would likely result in pulp exposure. The dentists were inquired whether they would approach the case differently. Those who answered “no” had their treatment option recorded as scenario I. For those who answered “yes”, their new treatment option was recorded. In both cases the prescription of antibiotics was also investigated.

### Sample size estimation

With 13.904 registered dentists in Greece in 2022 and based on the study by Stangvaltaite et al., it was expected that 50% of the respondents would manage DCLs with a desired precision of ± 5% and a 95% confidence interval and power of 0.8 (Stangvaltaite et al. 2013). Considering a nonresponse rate of 20% a minimum of 449 respondents was required.

### Data analysis

The responses were collected using the Google Drive Excel document (Microsoft Corp., Redmond, WA, USA). The data was imported into SPSS 28 (Inc., Chicago, IL, USA) and analyzed descriptively using the chi-squared and Fisher’s exact tests. For the cumulative analysis, selective caries removal, indirect/direct pulp capping and pulpotomy were all grouped as vital pulp treatment-VPT. The significance level was set at *p* < 0.05.

## Results

A total of 453 dentists completed the questionnaire. The response rate was estimated at 15%. The demographics of the respondents are presented in Table 1.

**Table 1:** Demographics of 453 respondents

Demographics	Mean % (*n*)
Gender	
Male	40.8 (185)
Female	58.7 (266)
Other	0.5 (2)
Education status	
DDs	60 (272)
MSc	33.6 (152)
PhD	6.4 (29)
Occupation	
Private practice	64.7 (293)
Dental clinic	20.1 (91)
NHS	5.5 (25)
Academic	9.7 (44)
Years of experience	
0–5 years	40 (181)
6–15 years	29.5 (134)
15 + years	30.5 (138)
Means of continuous education	
Multiple means	78.8 (357)
Seminars/congresses	18.5 (84)
Social media	0.9 (4)
Asking collegues	0.9 (4)
Journals	0.9 (4)
Total	100 (453)

**Fig. 3:**
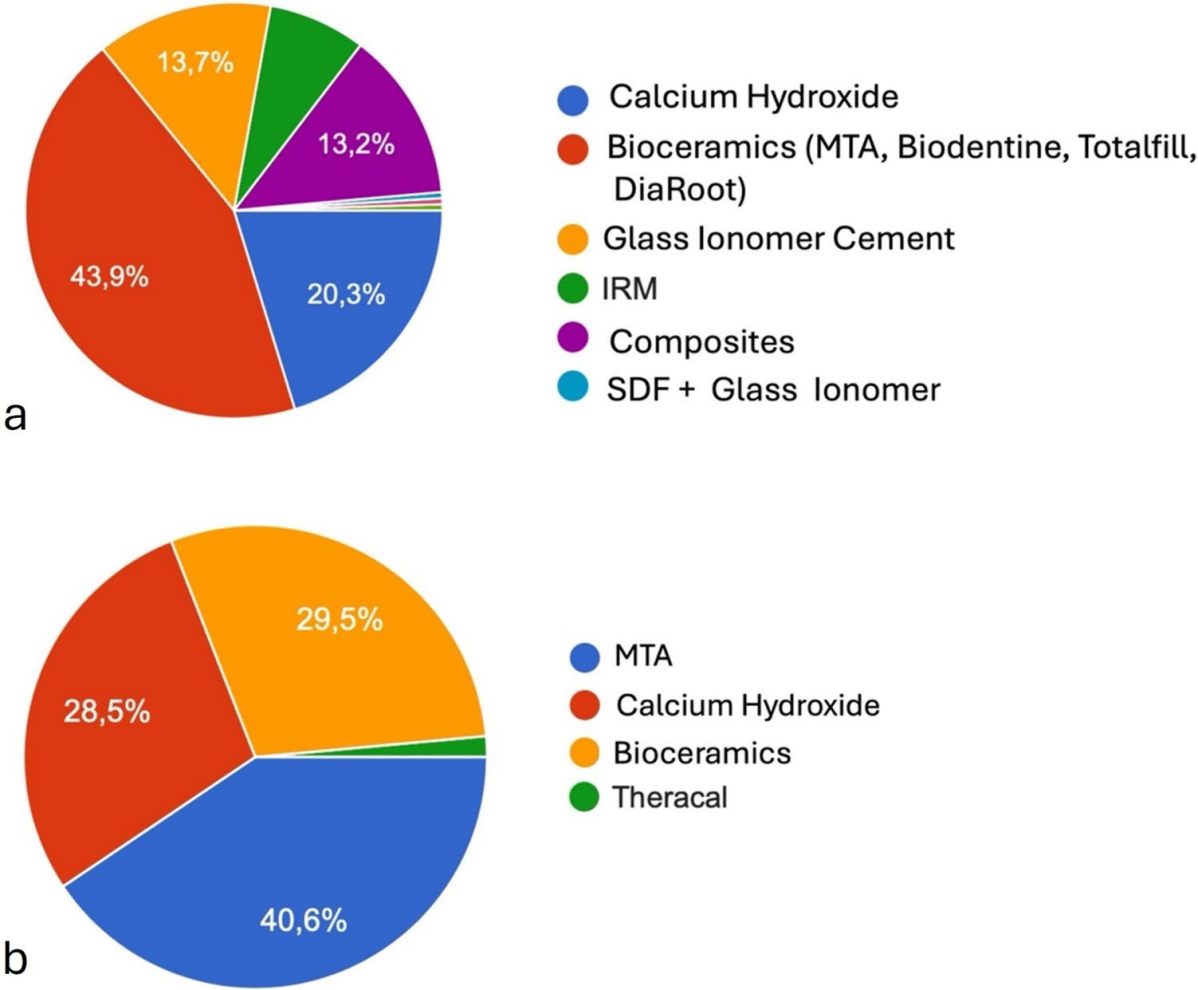
**a** Materials used for indirect pulp capping in scenario I. **b** Materials used for direct pulp capping and pulpotomy in Scenario I

In Scenario I, when confronted with a DCL on an asymptomatic mature tooth, 212 respondents (46.8%) chose a selective caries removal approach. The materials used for indirect pulp capping are presented in Fig. 3a. MTA and Bioceramics were the preferred choice when compared to all other materials (*p* < 0.01). 241 respondents (53.2%) chose total caries removal. In the case of pulp exposure where hemostasis is achieved within two minutes, the majority of the total caries removal respondents (199, 82.6%) chose direct pulp capping, 30 (12.4%) opted for root canal treatment and 8 (3.3%) chose pulpotomy. Four respondents provided an open answer representing 1.7% of the sample. Bioceramics and MTA were also the most preferred choice among all direct capping materials (*p* < 0.01) (shown in Fig. 3b).

In scenario II, treatment preferences of the selective caries removal group varied significantly when compared to all other groups (*p* < 0.05). Specifically, out of 453 respondents, 229 (50.6%) chose selective caries removal. Regarding the rest respondents, 159 (35.1%) preferred direct pulp capping, 50 (11%) selected pulpotomy, 13 (2.9%) opted for root canal treatment (RCT), while 2 (0.4%) would refer the patient to a specialist. In scenario II, 134 respondents (29.5%) consider a different approach compared to scenario I. Out of 134 dentists surveyed, 55 (41%) switched to selective caries removal with 43 of them originating from the direct pulp capping group and 12 from the RCT group of scenario I. Out of 134, 41 (30.6%) chose pulpotomy, with 13 of them initially originating from the selective group of scenario I and the remaining 28 from the direct pulp capping group of scenario I. Thirty-one (23.2%) out of 134 participants opted for direct pulp capping, with 24 coming from the selective group and 7 from the RCT group in scenario I. Finally, 5/134 (3.7%) preferred RCT and 2/134 (1.5%) would refer the patient to a specialist. Treatment preferences of the respondents who changed their treatment options in scenario II are presented in Table 2.

**Table 2:** Treatment preferences of the respondents who change their treatment option in the case of an immature tooth with signs and symptoms of reversible pulpitis

Treatment preference in Scenario II	Mean % (*n*)	Treatment preference in Scenario I
Selective caries removal	41 (55)	Direct pulp capping 32% (43) RCT 9% (12)
Pulpotomy	30.6 (41)	Direct pulp capping 19.4% (26) Selective caries removal 9.7% (13) RCT 1.5% (2)
Direct pulp capping	23.2 (31)	Selective caries removal 17.9% (24) RCT 5.3% (7)
Root canal treatment (RCT)	3.7 (5)	Direct pulp capping 3% (4) Selective caries removal 0.7% (1)
Other	1.5 (2)	RCT 0.75% (1) Direct pulp capping 0.75% (1)
Total	100 (134)	100% (134)

The first column includes the treatment preference selected for the immature tooth while the third column describes the treatment preference of the same group of respondents in scenario I (fully developed tooth)

Overall, treatment options in scenario II were not significantly different (*p* > 0.05) compared to those in scenario I, except for root canal treatment (*p* < 0.001). Cumulatively, most of the respondents; 423 (93.4%) for scenario I and 440 (97.1%) for scenario II preferred a VPT option.

The correlation between years of experience and treatment preference showed that dentists with 15 + years of experience tended to choose root canal treatment more often in scenario I (*p* < 0.05). According to logistic regression models, dental education or specialty training did not affect results. None of the other demographic variables were statistically associated with the treatment preferences in either scenario (Tables 3 and 4). Regarding antibiotic prescription, 9 out of 453 (2%) respondents chose to do so.

**Table 3:** Binary logistic regression analysis correlating demographic factors with responses in scenario I and II

	Scenario I								Scenario II			
	Caries removal		OR (95% CI)	*p*	Pulp capping		OR (95% CI)	*p*	Treatment change		OR (95% CI)	*p*
	Selective	Total			Yes	Other			Yes	No		
Gender												
Male	86	99	1		79	20	1		59	126	1	
Female	125	141	0.980 (0.673–1.427)	0.916	121	20	0.653 (0.330–1.291)	0.220	97	169	0.816 (0.548–1.214)	0.315
Other	1	1	0.869 (0.054–14.098)	0.921	1	0	0.000 (0.000)	1.000	1	1	0.468 (0.029–7.616)	0.594
Education												
DDs	135	137	1		116	21	1		94	178	1	
MSc	63	89	1.056 (0.491–2.271)	0.106	74	15	1.120 (0.543–2.309)	0.760	50	102	1.077 (0.707–1.641)	0.729
PhD	14	15	1.392 (0.932–2.078)	0.890	11	4	2.009 (0.584–6.908)	0.268	13	16	0.650 (0.300–1.408)	0.275
Occupation												
Private practice	135	158	1		131	27	1		102	191	1	
Dental clinic	44	47	0.913 (0.570–1.462)	0.704	40	7	0.849 (0.344–2.096)	0.723	36	55	0.816 (0.503–1.324)	0.410
NHS	12	13	0.926 (0.409–2.097)	0.853	11	2	0.882 (0.185–4.209)	0.875	8	17	1.135 (0.474–2.720)	0.777
Academic	21	23	0.936 (0.496–1.765)	0.838	19	4	1.021 (0.322–3.242)	0.971	11	33	1.602 (0.777–3.303)	0.202
Experience												
0–5	85	96	1		88	8	1		62	119	1	
6–15	65	69	0.940 (0.601–1.470)	0.786	59	10	1.864 (0.695–5.000)	0.216	42	92	1.141 (0.708–1.839)	0.587
15 +	62	76	1.085 (0.696–1.693)	0.718	54	22	4.481 (1.864–10.775)	< 0.001*	53	85	0.836 (0.527–1.324)	0.444
Continuous education												
Multiple means	168	189	1		159	30	1		123	234		
Seminars	40	44	0.978 (0.607–1.574)	0.926	36	8	1.178 (0.499–2.782)	0.709	30	54	0.946 (0.576–1.555)	0.827
Asking colleagues	3	1	0.296 (0.031–2.876)	0.294	1	0	0.000	1.000	2	2	0.526 (0.073–3.777)	0.523
Journals	1	3	2.667 (0.275–25.881)	0.398	2	1	2.650 (0.233–30.160)	0.432	2	2	0.526 (0.073–3.777)	0.523
Social Media	0	4	1.435.977.638 (0.0–0.0)	0.999	3	1	1.767 (0.178–17.560)	0.627	0	4	849.159.853.29 (0.0)	0.999

Selective caries and pulp capping removal are the reference variables for scenario I, tretamnet change is the reference variable for Scenario II

OR odds ratio

CI confidence interval

*p < 0.001

**Table 4:** Multivariate logistic regression analysis: model for correlating demographic variables with clinical decisions in immature teeth

Treatment	Demographics	B	S.E	Wald	df	Sig	Exp (*β*)	95% CI for Exp (*β*)	
								Lower	Upper
Direct pulp capping	Gender	0.407	0.443	0.844	1	0.358	1.503	0.630	3.583
	Education	0.029	0.339	0.007	1	0.932	1.029	0.530	2.000
	Occupation	0.311	0.270	1.331	1	0.249	1.365	0.805	2.317
	Experience	− 0.406	0.265	2.347	1	0.126	0.667	0.397	1.120
	Cont. educ	− 0.083	0.397	0.044	1	0.834	0.920	0.422	2.005
Pulpotomy	Gender	− 0.062	0.424	0.021	1	0.884	0.940	0.410	2.157
	Education	− 0.219	0.340	0.418	1	0.518	0.803	0.413	1.562
	Occupation	0.550	0.254	4.702	1	0.030**	1.734	1.054	2.851
	Experience	− 0.184	0.254	0.523	1	0.470	0.832	0.506	1.369
	Cont. educ	− 0.412	0.427	0.932	1	0.334	0.662	0.287	1.529
RCT	Gender	− 1.237	1.352	0.837	1	0.360	0.290	.021	4.108
	Education	2.598	1.253	4.303	1	0.038	13.437	1.154	156.47
	Occupation	0.368	0.550	0.447	1	0.504	1.445	.491	4.250
	Experience	− 22.998	0.000		1		1.029e	1.029e	1.029e
	Cont. educ	0.514	1.002	0.263	1	0.608	1.672	0.234	11.926
Other	Gender	1.081	1.611	0.451	1	0.502	2.949	0.126	69.275
	Education	0.401	1.205	0.111	1	0.739	1.493	0.141	15.845
	Occupation	− 18.782	0.000		1		6.964e	6.964e	6.964e
	Experience	− 0.359	0.802	0.201	1	0.654	0.698	0.145	3.363
	Cont. educ	1.791	0.913	3.843	1	0.05**	5.993	1.000	35.904

**p < 0.05

## Discussion

The present study aimed to investigate dentists’ decision-making processes regarding the implementation of selective caries removal and pulp exposure in cases involving immature and fully developed teeth displaying signs of reversible pulpitis. According to the results of this study, less invasive and conservative treatment options including selective caries removal have not been fully implemented into the everyday clinical practice of Greek dentists for managing reversible pulpitis. It has been indicated that treatment preferences are influenced by the tooth developmental status, vary significantly and there is no clear preference for a more conservative approach to preserving as much healthy pulp tissue as possible.

Surveys are a reliable tool frequently used to evaluate dentists’ knowledge, attitudes, and decision-making processes. The treatment preferences of dentists regarding DCLs have been the subject of many surveys in different countries, most of which have included various clinical scenarios of fully developed teeth presenting signs and symptoms of reversible pulpitis (Oen et al. 2007, Koopaeei et al. 2017, Crespo-Gallardo et al. 2018, Alnahwi et al. 2018, Stangvaltaite et al. 2013, Schwendicke et al. 2017, Chai et al. 2020, Croft et al. 2019, Jurasic et al. 2022, Bjørndal et al. 2010, Schwendicke et al. 2016, Maltz et al. 2018). Total caries removal was the prevalent choice among dentists in the USA (Oen et al. 2007; Koopaeei et al. 2017; Jurasic et al. 2022), Spain (Crespo-Gallardo et al. 2018), Saudi Arabia (Alnahwi et al. 2018), Norway (Stangvaltaite et al. 2013), France and Germany (Schwendicke et al. 2017). However, a more conservative approach like selective caries removal was preferred by dentists in Australia (Chai et al. 2020), Finland (Croft et al. 2019) and Norway (Schwendicke et al. 2017).

Dental caries in children and adolescents is a common issue for paediatric dentists (Stecksen-Blicks et al. 2004) with the posterior teeth being the most frequently affected (Mejare et al. 2004). Vital pulp treatment modalities represent viable options for treating DCLs in primary and immature permanent teeth (Duggal et al. 2022; Tong et al. 2022). The European Academy of Paediatric Dentistry (EAPD) guidelines recommend that in such cases the least invasive technique leading to the optimal result should be used (Duggal et al. 2022). According to the ESE position statement, selective carious-tissue removal is highly recommended for managing DCLs in mature or immature teeth with reversible pulp damage. The procedure indicates that soft or firm dentin is left on the pulpal aspect of the cavity, whilst peripheral carious dentin is removed to hard dentin to achieve ideal bonding and sealing of the defect (ESE 2019). The biological rationale behind selective caries removal lies in the fact that sealing off the lesion from the oral environment inactivates caries progression and induces the formation of reparative dentin avoiding pulp exposure, a fact having a significant impact on the long-term prognosis of the tooth and treatment costs (Ricketts et al. 2013, Schwendicke et al. 2013, Bjørndal et al. 2010). The International Caries Consensus Collaboration (ICCC) report also recommends selective caries removal (Schwendicke et al. 2016). These recommendations are also supported by clinical trials on mature teeth showing that selective caries removal resulted in a 60–80% five-year survival rate of the pulp compared to the 46% five-year survival rate in the non-selective removal group (Maltz et al. 2018; Bjørndal et al. 2017).

In contrast, the AAE position statement suggests total caries removal for all cases using caries detectors or laser fluorescence as adjunct tools irrespectively of the root maturation stage (Glickman et al. 2009, AAE 2021). Total caries removal and complete elimination of bacteria from dentin are essential for the healing process. This decision is based on the fact that any remaining bacteria in the firm dentin cavity floor by the selective carious removal method could be a source of pathogen-associated molecular pattern irritants that may induce persistent inflammatory effects on pulp tissue limiting the regenerative or reparative potential of the pulp (AAE 2021). The potential for pulp exposure is not considered a disadvantage since direct visualization of the pulp can assist in evaluating haemostasis and the level of inflammation. As a result, other VPT modalities such as direct pulp capping or partial pulpotomy present as equally effective alternatives. So far there is limited evidence about success rates of VPT procedures in immature teeth. In the meta-analysis of Tong et al. it has been concluded that there is no clear evidence of the superiority of a VPT technique regarding clinical and radiographic success rates of immature teeth (Tong et al. 2022). A total of 12 studies were finally reviewed, with one of them being a randomized clinical trial. VPT treatment modalities included selective (in one or two steps) or total carries removal, direct pulp caping and partial or total pulpotomy. Indirect pulp capping was evaluated only in one paper, a fact evidencing the lack of experimental data regarding the management of DCLs in immature teeth.

The results of the current study show that over half (53.2%) of dentists prefer total caries removal when treating a permanent mature tooth showing signs and symptoms of reversible pulpitis (Scenario I). This shows that Greek dentists are not clearly aware of the indications for selective caries removal and the ESE position statement is not fully implemented in clinical practice. In the case of pulp exposure, most dentists (82.6%) of the total caries removal group preferred direct pulp capping. Despite most dentists showing a preference for VPT procedures like pulp capping or pulpotomy, there is still a minority (12.4%) who chose root canal treatment. Perhaps this is due to a lack of training and the unfamiliarity of dentists with VPT and related materials (Mejàre and Cvek 1993). The absence of specific equipment could impact decision-making as magnification is necessary to ensure the complete removal of soft dentin and thorough inspection of pulp tissue (Chailertvanitkul et al. 2014).

In the case of immature teeth with symptoms of reversible pulpitis almost half of the respondents (50.6%) opted for indirect pulp capping, and 46.1% chose direct pulp capping or pulpotomy. Compared to scenario I, the increase in preference for VPT options is statistically significant (*p* < 0.001). This indicates that dentists tend to avoid RCT due to the complexity of cases involving immature roots. In these cases, VPT may be the only viable treatment option that ensures continuing normal root maturation and apical closure. In these cases, a pulpectomy without performing complex procedures, like apexogenesis or apexification, is not a viable option.

However, the preferred VPT options presented a significantly wide range of choices. Overall, less than half of the respondents chose a more conservative approach, while the rest selected a more aggressive treatment option. Specifically, 41% of the respondents shifted from the total caries removal treatment option (scenario I) to the selective caries removal treatment option in scenario II. This decision emphasizes the importance of preserving pulp vitality by using more conservative treatment options to avoid pulp exposure. Conversely, there was also a noticeable shift towards more aggressive VPT options, such as pulpotomy (30.6%) or direct pulp capping (23.2%), among most respondents who originated from the selective caries removal group in scenario I. This indicates that many dentists may not be familiar with treatment options for immature teeth and may instead prefer to choose a more aggressive VPT modality, as a more preventive step, when root canal treatment is not an option for an immature tooth.

A similar study showed that French dentists also discerned between mature and immature teeth; 77% of the respondents preferred a conservative approach to caries removal for permanent immature teeth compared to 21.5% for permanent mature teeth (Muller-Bolla et al. 2021). So far, the ESE and AAE guidelines do not differentiate between cases of fully developed and immature teeth. Therefore, the same guidelines applied for mature teeth with reversible and irreversible pulpitis were also applicable to immature teeth. However, the ESE S3 Level Guidelines indicate that VPT is the first clinical approach for immature teeth with signs and symptoms of pulpitis (symptomatic), whilst root canal therapy or full pulpotomy is suggested for mature teeth (Duncan et al. 2023). This study clearly shows that respondents discriminate between cases based on root maturation stage, indicating that teeth with immature apex present more complex cases.

Clinical decision-making for DCLs in immature permanent teeth should consider the specific clinical conditions found in children and adolescent teeth. Compared to mature permanent teeth, immature teeth have thinner and less mineralized dentin with increased permeability due to the wide dentinal tubules and large pulp chambers with higher pulp horns (Chowdhary et al. 2010). As these factors significantly increase the risk of pulp exposure it is imperative that diagnosis along with the thickness of the remaining dentin is the only key factor in determining the type of intervention needed. It has been indicated that to perform selective caries removal, a clearly defined radiopaque zone of dentin that is calculated no less than a quarter of the full dentin thickness should separate pulp from the cavity floor (ESE 2019). Unfortunately, calculating the remaining dentin thickness can be challenging in periapical radiographs. In such cases, the parallel x-ray technique, specific software tools of a digital imaging system, bitewings, radiograph subtraction as well as the use of magnification may be necessary for more accurate estimations (Kühnisch et al. 2020). Though the regenerative capacity of the young dental pulp of children teeth is superior compared to that of adults, it could not negate the fact that pulp exposure in cases of immature teeth is a prognostic factor that may negatively impact the success rates of VPT treatment. Considering the biological benefits of pulp preservation, the conservative approach to dentin removal is still imperative (Glickman et al. 2009). Consequently, the management of DCLs should be considered otherwise between child and adult patients.

Regarding the materials used for indirect or direct pulp capping, MTA and Bioceramics were the most preferred options. This choice aligns with the ESE guidelines (ESE 2019). These materials possess superior anti-inflammatory and anti-bacterial properties, as well as sealing ability and biocompatibility, when compared to traditional capping materials like calcium hydroxide (Chicarelli et al. 2021). Most importantly they present superior histological outcomes in terms of the quality of dentin bridge formation, which is directly correlated with improved clinical results (Nair et al. 2008; Kundzina et al. 2017). While Bioceramics present the gold standard for direct pulp capping it should be noted that there is no definitive evidence supporting a single indirect capping material.

Only 1.9% of dentists prescribed antibiotics. The use of systemic or topical antibiotics is not indicated in cases of reversible pulpitis or pulp exposure (Segura-Egea et al. 2018).

In this study, most dentists work in their own private practice. However, no statistically significant difference was found in treatment preferences between dentists working in private practices and those working in dental clinic settings. Most of the participants had 0–5 years of clinical experience (40%), indicating that they had graduated more recently and tended to prefer VPT with a statistically significant difference (*p* < 0.05) compared to the group with 15 + years of experience. This could be interpreted as a tendency of dental schools’ curricula to emphasize VPT in recent years. The preference for conservative treatment approaches, including selective caries removal or other VPT modalities, may also be influenced by the perceived novelty and contemporary nature of such methods. On the other hand, dentists with over 15 years of experience tend to prefer RCT, due to their increased confidence and familiarity with this established procedure. Moreover, the rapid dissemination of scientific knowledge through meetings, published statements, and official social media platforms makes updating the dental community quick and effective. In this study, most of the participants (78.8%) chose a combined method of continuing education through congresses, lectures, webinars and social media. Consequently, it is expected that guidelines expressed in official position statements would be widely integrated into the dental community. Perhaps, the discrepancy can be explained by attending low-quality webinars or social media sources that do not accurately represent the official endodontic communities. This highlights the need for educational initiatives within dental curricula or continuing education programmes to promote conservative treatment approaches for dealing with DCLs in Greece.

This study is the first to investigate Greek dentists’ preferences for managing deep carious lesions (DCLs) in mature and immature teeth. It addresses a gap in the literature regarding how clinical decision-making varies depending on root development. The questionnaire presents realistic clinical cases with radiographs and symptom descriptions, enhancing the validity of the responses. A total of 453 respondents participated with varying levels of experience and educational backgrounds, allowing for broader generalizability of the findings. Moreover, the sample size ensured a substantial dataset for meaningful statistical analysis. However, this study is not free from certain limitations. As forementioned, the questionnaire was distributed exclusively through digital channels and links, therefore the precise number of dentists reached could not be accurately determined. Other limitations of the current study include the sample which is not representative of the total dentist’s population and the fact that the information provided is based on self-reporting. Respondents may choose answers that seem theoretically correct but do not reflect their actual clinical practice. Everyday decision-making is affected by numerous factors. Closed-ended questions may not accurately represent real-life management decisions. Finally, specific VPT options like stepwise caries removal were not included in the questionary to clearly emphasize the main dilemma which is the selection between total or selective caries removal. Besides that, it has been shown that one-step selective caries removal yields the same, if not better, results than stepwise excavation since there are several studies recommending one-step selective excavation instead of a two-step (stepwise) procedure where there is still a possibility of pulp exposure during re-intervention (AAPD 2024, Maltz et al. 2018, Hoefer et al. 2016).

## Conclusion

Considering any limitation of the present cross-sectional questionnaire study investigating Greek dentists’ attitudes regarding conservative methods for caries removal and pulp capping procedures in immature and fully developed teeth with reversible pulpitis the following conclusions can be made:

- The absence of a clearly defined strategy and consensus between European and American (ESE/AAE) guidelines regarding the management of deep carious lesions in permanent teeth may have an impact on treatment decisions made within the Greek dental community.
- Though treatment preferences are influenced by the tooth developmental status, less invasive and conservative treatment options have not been fully implemented into everyday clinical practice.
- These findings highlight the necessity for the adoption of a unique, aetiology-related and age-dependent protocol for deep caries lesions management that would be commonly adopted and supported by dental practitioners.

## Data Availability

All data supporting the findings of this study are available within the paper.
